# Spontaneous Lumbar Artery Injury Resulting in Retroperitoneal Hematoma Mimicking Abdominal Aortic Aneurysm Rupture

**DOI:** 10.3400/avd.cr.21-00069

**Published:** 2021-12-25

**Authors:** Shingo Nakai, Tetsuro Uchida, Yoshinori Kuroda, Atsushi Yamashita, Eiichi Ohba, Masahiro Mizumoto, Jun Hayashi, Kimihiro Kobayashi, Tomonori Ochiai

**Affiliations:** 1 Second Department of Surgery, Yamagata University Faculty of Medicine, Yamagata, Yamagata, Japan

**Keywords:** spontaneous lumbar artery injury, retroperitoneal hematoma, abdominal aortic aneurysm rupture

## Abstract

A 73-year-old woman, who had previously undergone endovascular aortic repair (EVAR), developed severe back pain while shoveling snow. Preoperative computed tomography (CT) revealed marked retroperitoneal hematoma around the abdominal aortic aneurysm (AAA) with extravasation of contrast media. Intraoperative angiography demonstrated spontaneous lumbar artery injury (SLI). The bleeding lumbar artery was embolized using lipiodol, and deteriorated hemodynamics were stabilized. SLI is rare and can mimic the clinical symptoms and CT findings of AAA rupture. Vascular surgeons should focus on the status of the aneurysmal sac and the possibility of another retroperitoneal disease to determine appropriate treatment options, despite successful EVAR for AAA.

## Introduction

Spontaneous lumbar artery injury (SLI) is a rare disease, and many clinicians are unfamiliar with this entity, its clinical features, and specific findings from computed tomography (CT) imaging. SLI often shows retroperitoneal hematoma, and its CT findings resemble those of ruptured abdominal aortic aneurysm (AAA). Retroperitoneal hematomas are commonly caused by several well-recognized factors, including ruptured AAA, traumatic vascular injury, and retroperitoneal neoplasms.^1)^ Lumbar artery injury is also a common cause of retroperitoneal hematoma formation. Except for blunt traumatic accidents, lumbar arteries can be injured spontaneously, and SLI is a rare entity that causes retroperitoneal hematoma. Currently, only 15 cases of SLI have been reported.^1)^

Herein, we report a rare clinical course of SLI that resulted in retroperitoneal hematoma mimicking AAA rupture in a patient who had previously undergone endovascular aortic repair (EVAR) for AAA.

## Case Report

**Figure 1:**
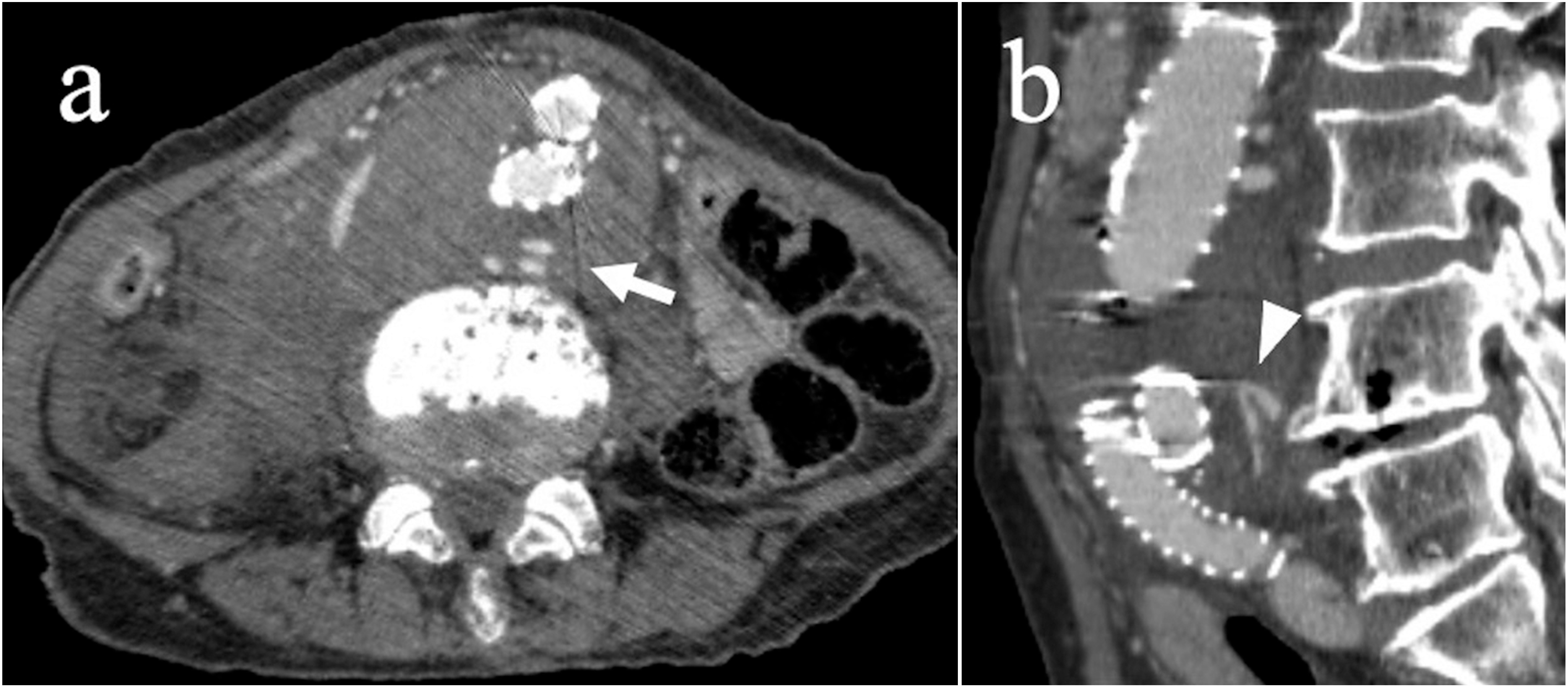
Fig. 1 (**a**) Axial view of CT imaging shows retroperitoneal hematoma around the sac of abdominal aortic aneurysm and extravasation of contrast media (white arrow). (**b**) Sagittal view of CT imaging shows a sharp spike formation on the lumbar vertebra adjacent to the area of extravasation (white arrowhead).

A 73-year-old woman, who presented with severe back pain while shoveling snow during heavy snowing, with shock status was transferred to our institution. The patient had undergone EVAR using Aorfix (Lombard Medical, Inc., Irvine, CA, USA) for a 53-mm AAA 3 years prior and was followed up regularly at the outpatient clinic of our institution. She had also received oral anticoagulant therapy for chronic atrial fibrillation. Follow-up CT performed a month earlier demonstrated adequate shrinkage of the aneurysmal sac to 30 mm around the endograft with no signs of endoleak. Soon after arrival at the emergency room, CT was performed again. A large amount of retroperitoneal hematoma was detected with extravasation of the contrast media (**Fig. 1a**). Sagittal CT imaging also showed sharp spike formation on the lumbar vertebra near the extravasation area (**Fig. 1b**).

CT: computed tomography

**Figure 2:**
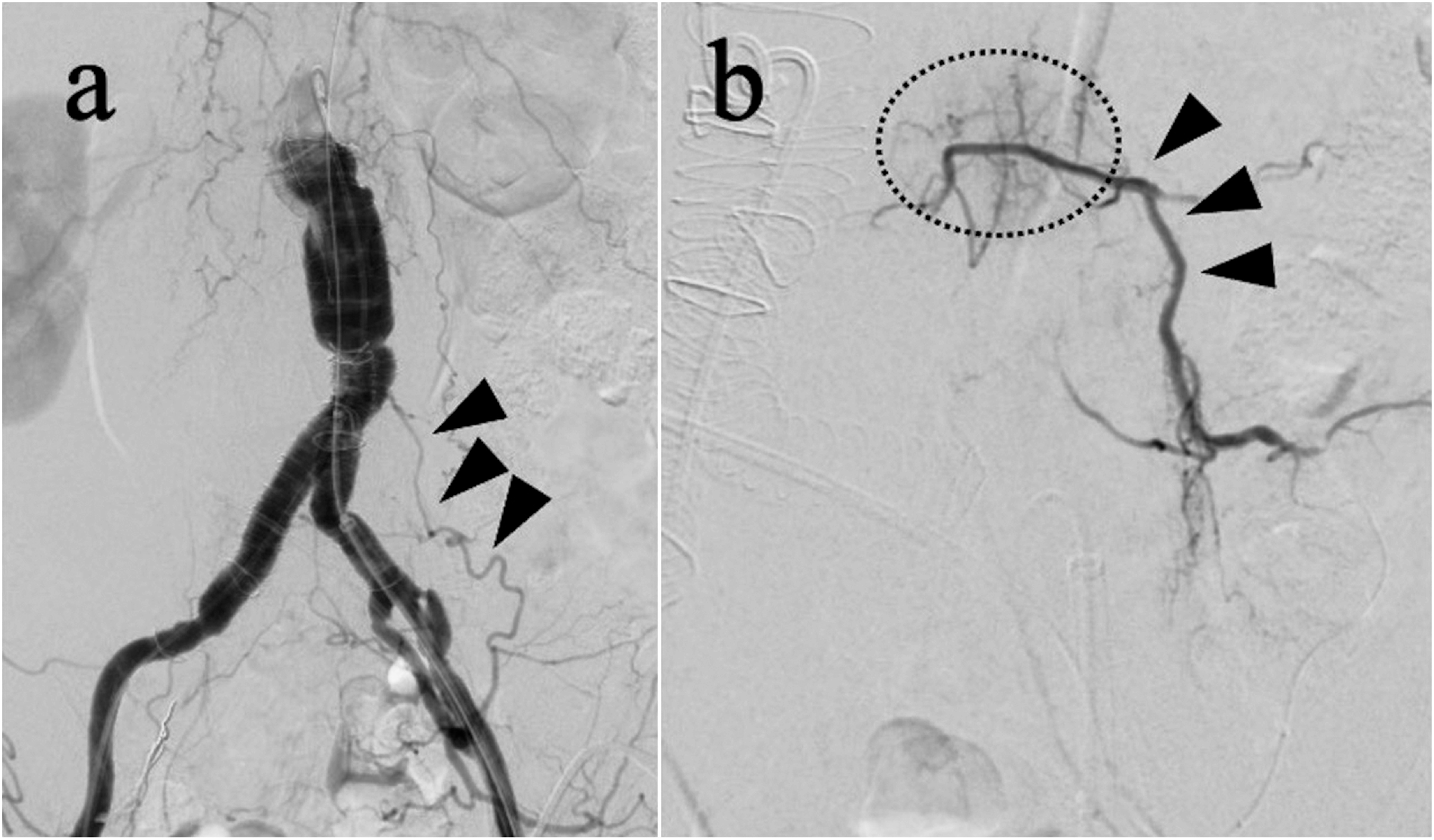
Fig. 2 (**a**) Intraoperative angiography reveals no endoleaks of any type that can cause aneurysm rupture (arrowheads). (**b**) The lumbar artery causing the bleeding in the retroperitoneal space is embolized using lipiodol (arrowheads). As in the CT, extravasation is detected in the lumbar artery at the level of the fourth lumbar vertebra (black circle).

Among several diagnoses to be considered, aneurysmal sac rupture owing to newly developed endoleaks was suspected as the most likely cause of retroperitoneal hematoma, although recent CT imaging confirmed aneurysmal sac shrinkage and no endoleak. Considering the possibility of another pathogenesis of retroperitoneal hematoma, after preparation of endovascular instruments including a stent graft, an angiography through the left femoral artery was performed to obtain a definitive diagnosis. Endoleak from the stent graft was also not detected, and the aneurysmal sac had not expanded or ruptured; therefore, ruptured AAA was ruled out. On the other hand, extravasation of the contrast media to the retroperitoneal space from the lumbar artery was confirmed (**Fig. 2a**). The patient was diagnosed with SLI, which might have been related to the snow shoveling (a difficult activity in winter conditions in Japan). Based on this diagnosis, the injured lumbar artery was embolized at the level of the fourth lumbar vertebra via the left internal iliac artery using lipiodol (**Fig. 2b**). The artery branching from the internal iliac artery was selected with three microcatheters, and the two lumbar arteries were embolized. Hemostasis was achieved, and the deteriorated hemodynamics was stabilized soon after the catheter embolization. The postoperative course was uneventful, and anticoagulation was not restarted postoperatively because there was no evidence of atrial fibrillation. Postoperative CT showed disappearance of the extravasation and volume reduction of the retroperitoneal hematoma.

CT: computed tomography

## Discussion

**Figure 3:**
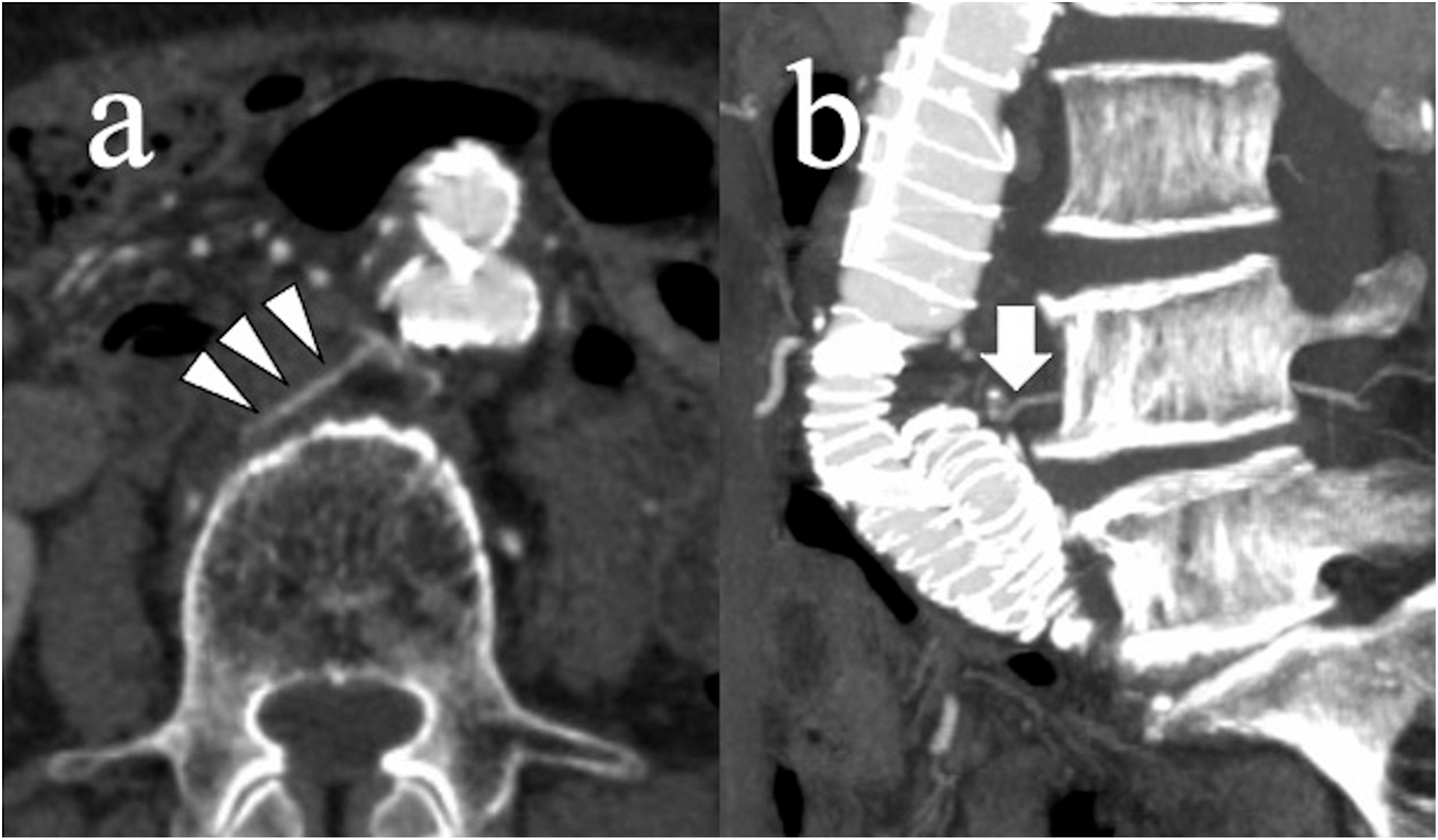
Fig. 3 (**a**) Overstretched lumbar artery due to shrinking abdominal aortic aneurysm (white arrowheads). (**b**) Protruding osteophyte of the fourth lumbar vertebra and stretched lumbar artery running nearby (white arrow).

The precise mechanism of SLI is unclear. However, advanced age and end-stage renal failure including renal transplantation, hemodialysis, and antiplatelet and anticoagulation therapy were reported as risk factors of SLI.^1,2)^ Among several pathophysiologies to be considered, prolonged supine position and overstrain of the iliopsoas muscle were reported as possible causes of SLI.^3)^ In our present case, the mechanism of SLI is considered multifactorial: (1) overstretching of the lumbar artery with shrinkage of the aneurysmal sac after successful EVAR (**Fig. 3a**), (2) further overstretching of the lumbar artery by overstrain of the iliopsoas muscle caused by repeated flexion and extension of the lumbar spine during snow shoveling, and (3) rubbing or scratching by the spike of the lumbar vertebra adjacent to the lumbar artery (**Fig. 3b**). Furthermore, retroperitoneal bleeding might be enhanced by oral anticoagulant therapy for atrial fibrillation.

Clinical symptoms of SLI include abdominal pain, back pain, and hemodynamic deterioration by retroperitoneal bleeding.^2)^ Furthermore, the major CT finding of SLI is retroperitoneal hematoma. With regard to differential diagnosis, these clinical features are similar to those of ruptured AAA. SLI resulting in retroperitoneal hematoma mimics AAA rupture, and these similarities make diagnosis difficult even for experienced vascular specialists. To make matters more complex, our present case had a history of EVAR for AAA. Therefore, we tended to focus on postoperative issues regarding the endograft itself and the aneurysmal sac on the patient’s arrival. Fortunately, angiography clearly revealed extravasation of the contrast media into the retroperitoneal space from the lumbar artery, and the diagnosis of SLI was confirmed. While treating emergency patients who present with retroperitoneal hematoma, vascular surgeons should keep in mind that SLI can mimic the clinical symptoms and CT findings of AAA rupture.

According to the literature, among patients treated surgically for spontaneous retroperitoneal hemorrhage, uncontrolled bleeding was reported in 4 out of 10 cases.^4)^ Open surgical hemostasis is not necessarily effective in all cases. On the other hand, transcatheter arterial embolization is the first line therapy for SLI in the recent endovascular era.^4,5)^ In our present case, angiography was performed for the definitive diagnosis after preparation of the additional EVAR devices. Subsequent embolization of the injured lumbar artery could be successfully achieved.

## Conclusion

Retroperitoneal hematoma caused by SLI is a rare but potentially fatal condition that mimics ruptured AAA. It is mandatory for vascular surgeons to focus not only on the status of the aneurysmal sac but also on the possibility of another retroperitoneal disease in patients, even after successful EVAR for AAA.
